# Aflatoxin levels, plasma vitamins A and E concentrations, and their association with HIV and hepatitis B virus infections in Ghanaians: a cross-sectional study

**DOI:** 10.1186/1758-2652-14-53

**Published:** 2011-11-11

**Authors:** Francis A Obuseh, Pauline E Jolly, Andrzej Kulczycki, John Ehiri, John Waterbor, Renee A Desmond, Peter O Preko, Yi Jiang, Chandrika J Piyathilake

**Affiliations:** 1 Department of Health Care Organization and Policy, School of Public Health, University of Alabama at Birmingham, Birmingham, Alabama, USA; 2 Department of Epidemiology, School of Public Health, University of Alabama at Birmingham, Birmingham, Alabama, USA; 3 Division of Health Promotion Sciences, Mel and Enid Zuckerman College of Public Health, University of Arizona, Tucson, Arizona, USA; 4 Division of Preventive Medicine, School of Medicine, University of Alabama at Birmingham, Birmingham, Alabama, USA; 5 St. Markus Hospital, AIDS ALLY, Kumasi, Ghana; 6 Department of Nutrition Sciences - Nutritional Biochemistry and Genomics, University of Alabama at Birmingham, Birmingham, Alabama, USA

## Abstract

**Background:**

Micronutrient deficiencies occur commonly in people infected with the human immunodeficiency virus. Since aflatoxin exposure also results in reduced levels of several micronutrients, HIV and aflatoxin may work synergistically to increase micronutrient deficiencies. However, there has been no report on the association between aflatoxin exposure and micronutrient deficiencies in HIV-infected people. We measured aflatoxin B_1 _albumin (AF-ALB) adduct levels and vitamins A and E concentrations in the plasma of HIV-positive and HIV-negative Ghanaians and examined the association of vitamins A and E with HIV status, aflatoxin levels and hepatitis B virus (HBV) infection.

**Methods:**

A cross-sectional study was conducted in which participants completed a demographic survey and gave a 20 mL blood sample for analysis of AF-ALB levels, vitamins A and E concentrations, CD4 counts, HIV viral load and HBV infection.

**Results:**

HIV-infected participants had significantly higher AF-ALB levels (median for HIV-positive and HIV-negative participants was 0.93 and 0.80 pmol/mg albumin, respectively; p <0.01) and significantly lower levels of vitamin A (-16.94 μg/dL; p <0.0001) and vitamin E (-0.22 mg/dL; p <0.001). For the total study group, higher AF-ALB was associated with significantly lower vitamin A (-4.83 μg/dL for every 0.1 pmol/mg increase in AF-ALB). HBV-infected people had significantly lower vitamin A (-5.66 μg/dL; p = 0.01). Vitamins A and E levels were inversely associated with HIV viral load (p = 0.02 for each), and low vitamin E was associated with lower CD4 counts (p = 0.004).

**Conclusions:**

Our finding of the significant decrease in vitamin A associated with AF-ALB suggests that aflatoxin exposure significantly compromises the micronutrient status of people who are already facing overwhelming health problems, including HIV infection.

## Background

Sub-Saharan Africa accounts for approximately two-thirds of all persons infected by HIV, and approximately 70% of new cases of HIV infection worldwide [1]. Although the estimated adult HIV seroprevalence rate in Ghana in 2007 was 1.9% [2], the HIV sentinel survey indicates that the seroprevalence rate in the country varies by region from 0.8 to 8.4% [2].

Sub-Saharan Africa is disproportionately burdened by malnutrition and deficiencies of nutrients, such as vitamins A, B, C, D and E, which have been implicated in HIV transmission and progression [3-5]. These and other studies have shown that deficiencies of vitamins A and E are positively associated with HIV transmission, disease progression and mortality [3-7]. Micronutrient malnutrition further impairs the immune system by suppressing immune function necessary for survival [8].

A study by Jiang *et al *[9] showed that vitamin A deficiency is common in certain parts of Ghana and is associated with impairment of innate and cytotoxic immune function. Vitamin E is a lipophilic antioxidant that also protects cell membranes. Studies conducted in HIV-infected individuals have shown that vitamin E reduces the production of oxidant compounds in lymphocytes that would otherwise lead to viral activation or cell death [10]. Vitamin E deficiency has been shown to increase the occurrence of wasting, oxidative stress and HIV viral load, and is a driving force for viral mutation [11].

However, supplementation of vitamins A and E or multivitamins has not always been shown to have beneficial effects. For example, it has been shown that vitamin A may increase sexual or perinatal transmission of HIV by increasing genital shedding [12] or increase transmission through breast milk when breastfeeding mothers are supplemented [13]. Similarly, vitamin A supplementation trials have had mixed effects on clinical outcomes, such as child morbidity and HIV disease progression [14,15]. In addition, vitamin E may facilitate HIV entry into cells and higher plasma vitamin E levels have been associated with adverse outcomes in HIV [16].

Aflatoxins are toxic metabolites of *Aspergillus *species of fungi, such as *A. flavus *and *A. parasiticus*, which are found naturally in some staple foods, such as groundnuts, maize and other oil seeds. They constitute the most potent hepatocarcinogens known [17]. In West African countries, including Ghana, aflatoxins are commonly found as contaminants in human and animal food [18-21]. Crops can become contaminated with aflatoxin-producing fungi during growth, but fungal proliferation and toxin production increase during storage of improperly dried grains and nuts under hot, humid and unsanitary conditions.

Acute and chronic exposures to aflatoxins compromise immunity and enhance macro- and micro-nutrient malnutrition and neonatal jaundice [19,22,23]. Exposure to aflatoxin has been found to be associated with reduced serum concentrations of vitamins A and vitamin E in swine [24,25]. Two recent studies have reported on the association between aflatoxin B_1 _albumin (AF-ALB) adduct levels and vitamins A and E in Ghanaians.

Obuseh *et al *[26] found a significant inverse relationship between AF-ALB and vitamin A and a non-significant inverse relationship between AF-ALB and vitamin E deficiency, whereas Tang *et al *[27] found significant negative correlations between both vitamins A and E concentrations and AF-ALB levels. Jiang *et al *[28] also found alterations in certain immunological parameters of Ghanaians with high AF-ALB levels. These alterations could result in impairments in cellular immunity that decrease resistance to infections.

Thus, aflatoxin and HIV may work synergistically in HIV-positive people to increase micronutrient deficiencies and immune suppression, and so promote HIV disease progression. No studies have examined the association between micronutrient deficiency and aflatoxin exposure among people living with HIV. We measured aflatoxin levels and vitamins A and E concentrations in plasma of HIV-positive and HIV-negative Ghanaians chronically exposed to aflatoxin in their diets and examined the association of vitamins A and E concentrations, HIV status, AF-ALB levels and hepatitis B virus (HBV) infection.

## Methods

### Study location, design and target population

A cross-sectional study using a convenience sample of HIV-positive and HIV-negative males and females 19 years of age and older was conducted in Kumasi (a major maize and peanut-producing and consuming area) in the Ashanti Region of Ghana. All HIV-positive and some HIV-negative study participants were recruited from a hospital that cared for both HIV-positive and HIV-negative persons. Potential participants were introduced to the research team by the physicians. All HIV-positive persons who were not acutely ill were eligible to participate in the study. No participant was hospitalized or was acutely ill; all were outpatients.

Some HIV-negative persons were recruited from the community and all (clinic and community recruits) had no record of HIV positivity or symptoms of HIV infection (either from clinic records or self-report). HIV-negative individuals who were recruited from the community came to the hospital to participate in the study. HIV-positive study participants had previously been tested for HIV and their positive test results were available in their medical charts. Two rapid tests are used to screen for HIV in Ghana.

At the time of the study, the Determine HIV-1/2 test (Abbott Laboratories, Abbott Park, IL, USA) was used as the first screening test. If a person tested positive for HIV or had an indeterminate result, the result was checked using a RapiTest HIV 1 and 2 kit (Morwell Diagnostics GmbH, Egg/ZH, Switzerland). An ELISA test was used as a tiebreaker if there was disagreement in the results from the two rapid tests. Plasma samples from HIV-negative participants were tested for HIV using the Coulter p24 antigen assay and those found to be HIV negative were included as HIV negatives in the study. Approximately 30% of HIV-positive participants were on ART.

All participants were volunteers and gave informed consent. Pregnant women, individuals younger than 19 years of age and acutely ill persons were excluded from the study. A target sample size of 300 subjects was specified for the study. This sample size of 300 was based on the expected prevalence of vitamin A deficiency of 35%, an alpha level of 0.05 and precision of 5%. Based on a hypothesized difference of 25% (35% deficiency among HIV-negative and 50% among HIV-positive individuals), we would need about 150 per group to detect a statistically significant result (odds ratio of 2.0).

Informed consent was obtained from 305 (147 HIV-negative and 158 HIV-positive) participants who were enrolled in the study. Approval for the study was obtained from the Institutional Review Board at the University of Alabama at Birmingham (UAB), and the Committee on Human Research, Publication and Ethics, Kwame Nkrumah University of Science and Technology (KNUST) College of Health Sciences, Kumasi, Ghana.

### Data and blood sample collection

An interviewer-administered questionnaire on demographic characteristics was completed for each participant. A 20 mL sample of venous blood was drawn from each participant using sterile needles and vacutainer tubes. The tubes were wrapped in foil to reduce the effect of oxidation and light on retinol. Blood was transported to the laboratories of the Kumasi Center for Collaborative Research (KCCR) in Tropical Medicine at KNUST within six hours of collection.

Plasma was obtained by centrifugation at 3000 rpm for five minutes and aliquoted into vials for the different analyses, mainly retinol, tocopherol, HIV viral load, HBV surface antigen, and AF-ALB. The vials containing plasma for retinol and tocopherol analysis were wrapped in aluminum foil and kept in thick black polythene bags at -80°C. These samples were subsequently air transported to UAB and kept at -80°C until analyzed.

### Simultaneous determination of retinol (vitamin A) and tocopherol (vitamin E) in plasma

A modified version of the high-performance liquid chromatography (HPLC) procedure developed by Stacewicz-Sapuntzakis *et al *[29] was used to measure both vitamins A and E in plasma. The HPLC system included 150 × 3.9 mm Nova-pak C18 (4 microns) column with a guard pak pre-column (both from Waters, Milford, MA), Waters Millipore TCM column heater, Waters 490 multi-wavelength detector, Hitachi 655-61 processor, Hitachi 655A-11 liquid chromatography, and Bio-Rad auto sampler AS-100. The mobile phase consisted of methanol/acetonitrile/methylene chloride (50:45:5, v/v/v; Mallinckrodt Specialty Chemical Co., Paris, KY) run at 1 ml/min.

Vitamin A (all trans retinoic acid) was obtained from Sigma Chemical Co., St. Louis, MO, and vitamin E (dl-alpha tocopherol) and tocol were obtained from Hoffmann-La Roche Inc., Nutley, NJ. Tocol is a tocopherol derivative that is used as an internal standard to correct for any loss in retinol and tocopherol during the extraction procedure. It was chosen as an internal standard because it is well separated from retinol under the normal phase conditions. In preparation of the standards, vitamins A and E were dissolved in ethanol and concentrations were measured at 325 nm and 292 nm, respectively, using a programmable multi-wavelength detector (Waters 490). Tocol was dissolved in ethanol (0.3µg/mL). All procedures were performed in subdued yellow light. Fresh standards were prepared for each assay and standard curves were constructed by plotting peak heights against the concentrations of vitamin standards.

Plasma samples from study participants were thawed and 200 μL of each placed in a separate test tube; 100 μL of the internal standard (tocol) and 100 μL ethanol for protein precipitation were added and the tubes were vortexed for two minutes. For extraction, 1 mL of hexane (EM Science, Cherry Hill, NJ) was added and the mixture was vortexed for five minutes and centrifuged at 8000 revolutions per minute for 10 minutes. The top hexane layer containing the micronutrients was carefully removed with a Pasteur pipette into another microcentrifuge tube, dried using a rotary speed-vac concentrator/evaporator (Savant Instrument Inc, Farmingdale, NY), and heated to 37ºC for 25 minutes. The residue was dissolved in 200µL mobile phase and vortexed for 30 seconds. Twenty microliters of this extract was injected for chromatographic analysis.

Tocol internal standard was used to determine the percent recovery in samples. For quality control, pooled normal human plasma samples were divided into two portions of high and low concentration for vitamin A and E, and prepared for analysis in the same manner as the patient samples. These were run in each assay. Evaluation of the laboratory performance was assessed by comparing the results of the quality control samples with the mean and standard deviations (SD) calculated from the results of several runs of the assay. The run was rejected if any value fell outside the range of ± 2 SD from the mean.

### Determination of AF-ALB levels in plasma by radioimmunoassay

AF-ALB levels in plasma from study participants were determined by radioimmunoassay (RIA) [30]. The assay measures aflatoxin that is covalently bound in peripheral blood albumin and reflects aflatoxin exposure in the previous two to three months. Plasma samples were concentrated by high-speed centrifugal filtration, and the concentrated protein was re-suspended in phosphate buffered saline (PBS). Plasma albumin was determined by using a bromocresol purple dye binding method (Sigma, St. Louis, MO), and the amount of total protein was determined by using the Bradford procedure (San Rafael, CA). Total protein per sample was then digested with Pronase (Calbiochem, La Jolla, CA), and bound aflatoxin was extracted with acetone.

The RIA procedure [30] was used to quantify AF-ALB in duplicate plasma protein digests that each contained 2 mg of protein. Normal human serum/plasma samples purchased from Sigma-Aldrich (St. Louis, MO) and authentic AFB-albumin standard were used for quality control purposes. The standard curve for the RIA was determined by using a nonlinear regression method. The concentrations of albumin, total protein and AF-ALB in individual plasma samples were calculated, and the values were expressed as pmol AF-ALB per mg albumin [30]. The accuracy of the analysis based on three days ranged from 93.3% to 96.3% for low concentration quality control. (0.1 pmol AF-ALB) and from 92.2% to 97.3% for high concentration quality control (2 pmol AF-ALB). The within day imprecision was 5.9% (n = 15) for LQC and 2.9% (n = 15) for HQC. The overall variation of inaccuracy and imprecision rates are within 10%. The average recovery (0.1-5.0 pmol AF-ALB) was 88.1% ± 5.2%. The detection limit of the assay was 0.01 pmol/mg albumin.

### Determination of CD4+ T cell count

Circulating CD4+ T cell populations were determined by flow cytometry using fluorescein isothiocyante-labelled monoclonal antibody against CD4 (BD PharMingen, San Diego, CA). Isotype-matched controls (BD PharMingen, San Diego, CA) were used in all experiments. Briefly, cells were washed and stained with monoclonal antibodies for 30 minutes in the dark at 4°C. They were then washed twice with staining PBS supplemented with 0.1% sodium azide and 1% fetal bovine serum pH 7.4, (BD PharMingen, San Diego, CA) and fixed in 4% paraformaldehyde in PBS (BD PharMingen, San Diego, CA). The cells were subsequently run on a fluorescent activated cell sorting instrument (Becton Dickinson, San Diego, CA) and analyzed using CellQuest software. Cells were gated on live peripheral blood lymphocyte population identified by forward- and side-scatter parameters, and at least 10,000 cells were acquired. Absolute CD4 counts were derived by using the percentage of CD4+ T cells in relation to the lymphocyte fraction determined by automatic differential blood count, as performed in the biochemistry laboratory at the KNUST.

### Quantitative HIV-1 RNA assay

HIV-1 RNA was measured using a quantitative reverse transcriptase polymerase chain reaction assay (Amplicor Monitor, Roche Diagnostic System, Brandersburg, NJ). Virus from 0.2 ml of plasma was lysed in the kit lysis buffer, and the HIV RNA was precipitated using isopropanol and pelleted by centrifugation. After washing with ethanol, the RNA was re-suspended in the kit dilution buffer. Extracted RNA was amplified and detected according to the manufacturer's instructions. The results were reported as HIV RNA copies/mL. All undetectable values (below 400 copies) were assigned a value of 399. The maximum detectable limit was 750,000 copies/mL.

### Test for HBV surface antigen

HBV surface antigen (HBsAg) in plasma samples was determined using the Bio-Rad Enzyme Immunoassay according to the manufacturer's directions (Bio-Rad, Redmont, WA, USA). Briefly, 100µL of specimens or controls were added in duplicate to appropriate wells on a microwell strip plate coated with mouse monoclonal antibody to HBVsAg and incubated for 60 minutes at 37°C. After washing, 100µL of peroxidase-conjugated mouse monoclonal antibodies against HBsAg was added to each well and the plate was incubated for 60 minutes at 37°C.

The plates were then washed; 100µL of tetramethylbenzidine substrate solution was added to each well and incubated in the dark for 30 minutes at room temperature. The reaction was stopped with the addition of 100µL of stopping solution to each well and the plate was read on a spectrophotometer at 450 nm. A sample was considered initially reactive for anti-HBs if the absorbance value was greater than or equal to the cut-off value. The cut-off value was determined by addition of 0.07 to the mean absorbance value of the HIV-negative controls. Positive samples were determined by repeated reactivity in duplicate tests.

### Tests of liver function (aminotransferases, bilirubin, total blood protein and plasma albumin)

Hepatic function tests were conducted on plasma from participants at the UAB Hospital Laboratory. This included tests of the liver enzymes aspartate aminotransferase (AST) and alanine aminotransferase (ALT), liver transport (direct bilirubin), and liver synthesis (albumin and total protein). The normal range values were based on those in the University of Alabama Hospital Laboratories Bulletin of Information (revised October 2002).

### Statistical analysis

Categorical variables were compared using chi-square tests. The World Health Organization's generally accepted cut-off values for micronutrient deficiencies for retinol (20µg/dL) and tocopherol (0.5 mg/dL) were used to categorize participants as deficient (low) or normal (high) [31]. These cut-off points were based on tissue concentrations low enough to cause adverse health outcomes. Univariate comparisons among strata for continuous variables such as micronutrients, aflatoxin, total protein, viral load and CD4 cell count values, were evaluated by using the Wilcoxon rank sum test. Associations between continuous variables were assessed using the Spearman correlation coefficient.

A subset analysis was restricted to HIV-positive individuals and stratified based on the viral load and CD4+ cell counts. We quantified the relationship between aflatoxin and micronutrients by log (natural log) of the HIV viral load (high and low viral load based on the median cut-off point of 7.7 copies/mL) and CD4 counts (<200 cells/mm^3^, 200-499 cells/mm^3^, and >499 cells/mm^3^). CD4 was categorized based on the 1993 revised classification system by the Centers for Disease Control and Prevention [32].

The viral load categorization was based on published research that showed that people with viral loads below 10,000 copies/mL of blood did not show disease progression in greater than a nine-year period compared with people with higher viral loads [33]. The median viral load is high. The study was done before the recommendation was made to begin ARV treatment at a CD4 count of 350 cells/mm^3^. Therefore, antiretroviral (ARV) treatment was started at CD4 levels of 250 cells/mm^3^. Approximately 30% of study participants were on ARV treatment.

Multivariate linear regression was used to assess the relationship between levels of vitamins A and E as outcomes, and aflatoxin, HIV status and HBV as primary exposures of interest. Variables that were significant in the univariate analysis at p <0.10 or less were considered for multivariate analysis. To maintain the precision of our estimates, we normalized our exposure and outcome variables where necessary with a transformation to ensure model fit. Regression diagnostics, such as residual checking, were used to refine the model. We controlled for potential confounders, such as age, sex, occupation and education. All hypothesis tests were two tailed, with a Type 1 error rate fixed at 5%. All statistical analyses were performed with Statistical Analyses System version 9.1 SAS Institute Inc., Cary, North Carolina.

## Results

Table 1 presents the descriptive statistics for the 305 study participants by HIV status (147 HIV negative and 158 HIV positive). There was no significant difference in age between the two groups. The mean age ± standard deviation (SD) for HIV-negative participants was 39.0 ± 16.2 years and for HIV-positive participants was 38.7 ± 9.2 years. Sixty-six percent of HIV-positive participants and 60% of HIV-negative participants were younger than 40 years. There were significant differences (p <0.05) between the two groups with regard to sex, education and occupation. A higher percentage of HIV-positive than HIV-negative participants (67% versus 46%, respectively) were women, and HIV-positive participants were more likely than HIV-negative participants to be educated (87% versus 52%, respectively). Half of HIV-positive participants were traders, whereas half of HIV-negative participants were farmers.

**Table 1:** Descriptive statistics of the study population by HIV status

Characteristics	HIV- [N = 147] n (%)	HIV+ [N = 158] n (%)	P value
**Age (years)**			0.28
19-39	88 (59.9)	104 (65.8)	
≥ 40	59 (40.1)	54 (34.2)	
**Sex**			0.0004
Male	79 (53.7)	53 (33.5)	
Female	68 (46.3)	105 (66.5)	
**Formal education**			<.0001
No	70 (48.3)	21 (13.4)	
Yes	75 (51.7)	136 (86.6)	
**Intake of alcohol**			0.03
No	102 (76.1)	129 (86.0)	
Yes	32 (23.9)	21 (14.0)	
**Occupation**			<0.0001
Farmer	71 (50.3)	2 (1.6)	
Trader	25 (17.7)	61 (49.6)	
Farmer/trader	14 (10.0)	0 (0.0)	
Other	31 (22.0)	60 (48.8)	
**Knowledge of aflatoxin**			0.09
No	107 (83.6)	116 (75.3)	
Yes	21 (16.4)	38 (24.7)	
**Hepatitis B virus infection**			0.21
No	123 (84.3)	139 (89.1)	
Yes	23 (15.7)	17 (10.9)	
**Vitamin A ( μg/dL)**			<.0001
Low (<20 μg/dL)	46 (31.7)	124 (83.2)	
High (≥20 μg/dL)	99 (68.3)	25 (16.8)	
**Vitamin E (mg/dL)**			0.001
Low (<0.5 mg/dL)	106 (73.1)	131 (87.9)	
High (≥0.5 mg/dL)	39 (26.9)	18 (12.1)	
**Aflatoxin B1 (pmol/mg albumin)**			0.01
Low (<0.8 pmol/mg albumin)	85 (58.2)	68 (43.6)	
High (≥0.8 pmol/mg albumin)	61 (41.8)	88 (56.4)	
**HIV viral load **(log copies/mL)			
**Mean ± standard deviation**		8.4 ± 2.7	
**Median **		7.7	
**CD4 T cell counts **(cells/mm^3^)			
**Mean ± standard deviation**		308 ± 253	
**Median**		253	

There was no significant difference in HBV infection between the groups. Significant differences were noted in micronutrient status between the groups. Significantly higher percentages of individuals with low vitamin A (<20 µg/dL) and low vitamin E (<0.5 mg/dL) levels were HIV-positive (83% and 88%, respectively) compared with HIV-negative participants (32% and 73%, respectively). There were significant differences in the plasma concentration of aflatoxin; 56% of HIV-positive individuals had high levels of AF-ALB (≥0.8 pmol/mg albumin) compared with 42% of HIV-negative individuals. The mean CD4 count for HIV-positive participants was 308 ± 253 cells/mm^3 ^(median 253 cells/mm^3^), and the mean log viral load was 8.4 ± 2.7 (median 7.7).

The mean ± the standard deviation (SD) and median concentrations of micronutrients, AF-ALB and liver function tests for HIV-negative and HIV-positive participants are shown in Table 2. Vitamins A and E and AF-ALB concentrations were all significantly different (p < 0.01) between the two groups. The median level of vitamin A in HIV-negative participants was significantly higher than that of HIV-positive participants (27.5µg/dL versus 12.6µg/dL, respectively). Also, the median level of vitamin E in HIV-negative participants was significantly higher than that of HIV-positive participants (0.37 versus 0.24 mg/dL, respectively).The median AF-ALB level for HIV-positive participants was 0.93 pmol/mg albumin, and that for HIV-negative participants was 0.80 pmol/mg albumin (p <0.01). CD4 counts were not determined for the HIV-negative participants.

**Table 2:** Univariate statistics and distributions of vitamins A and E and plasma aflatoxin by HIV status

	HIV negative		HIV positive		
Variables	Mean ± SD	Median	Mean ± SD	Median	P value
**Micronutrients**					
Vitamin A (µg/dL)	32.4 ± 20.6	27.5	13.7 ± 7.5	12.6	<0.0001
Vitamin E **(**mg/dL)	0.4 ± 0.3	0.4	0.3 ± 0.2	0.2	<0.0001
**Aflatoxin B_1 _albumin adducts ** (pmol/mg albumin)	0.9 ± 0.5	0.8	1.1 ± 0.6	0.9	0.01
**Liver function tests**					
**Alanine aminotransferase **	17.9 ± 9.7	15.0	25.9 ± 17.8	21.0	<0.0001
(NR = 6-45U/L)					
**Aspartate aminotransferase**	41.3 ± 26.5	37.0	65.1 ± 66.4	53	<0.0001
(NR = 0-37U/L)					
**Bilirubin direct**	0.14 ± 0.08	0.1	0.15 ± 0.23	0.1	0.03
(NR = 0.1-0.3 mg/dL)					
**Albumin**	3.53 ± 0.44	3.6	3.21 ± 0.79	3.3	<0.0001
(NR = 3.4-5.0 g/dL)					
**Total protein**	7.34 ± 0.86	7.4	8.47 ± 1.63	8.4	<0.0001
**(**NR = 6.0-7.9 g/dL)					

The Wilcoxon rank sum test for equality of medians was conducted

Liver function tests (ALT, AST, direct bilirubin, albumin and total protein) differed by HIV status. AST and total protein were significantly higher among HIV-positive participants. Although ALT was significantly higher and albumin was significantly lower for the HIV-positive group, these values were within the normal ranges.

The subset analysis of HIV-positive individuals stratified by viral load and CD4 counts is shown in Table 3. The median micronutrient concentrations of vitamins A and E differed significantly by viral load. HIV-positive individuals with high viral loads had significantly (p <0.02) lower vitamin A or E concentrations than those with low viral loads. Stratification by CD4 counts showed that lower plasma vitamin A and E levels were associated with lower CD4 cell counts or more advanced immunosuppression. However, the difference was significant only for vitamin E (p = 0.004).

**Table 3:** Micronutrient concentrations in relation to HIV viral load and CD4+ T cell counts

	Vitamin A concentration (µg/dL)			Vitamin E concentration (mg/dL)		
	
	Mean ± SD	Median	P value	Mean ± SD	Median	P value
**Low viral load **(<7.7 log)	12.36 ± 7.42	13.99	0.02	0.22 ± 0.20	0.29	0.02
**High viral load **(≥7.7 log)	15.11 ± 7.37	11.58		0.29 ± 0.20	0.18	
**CD4 <200 **cells/mm^3^	12.60 ± 8.50	11.60	0.40	0.18 ± 0.16	0.11	0.004
**CD4 200-499 **cells/mm^3^	14.00 ± 6.50	13.30		0.28 ± 0.18	0.27	
**CD4 >499 **cells/mm^3^	15.75 ± 8.66	16.37		0.34 ± 0.27	0.31	

The Wilcoxon rank sum test for equality of medians was conducted

There was no significant difference in AF-ALB concentration according to viral load or CD4+ T cell count. Spearman's correlation coefficients between variables showed significant correlations for AF-ALB with vitamin A (r = -0.20, p = 0.0007). Also, although vitamin E was not significantly correlated with AF-ALB, vitamins A and E were strongly correlated (r = 0.50, p <0.0001). When liver function concentrations within the HIV-positive group were stratified by viral load and CD4 count, there were no striking differences. Therefore, liver function data were not included in the multivariate analysis.

### Regression analysis

We found a significant negative relationship between AF-ALB and vitamin A concentration (p <0.01) (Table 4). Higher aflatoxin exposure was associated with lower vitamin A (-4.83 μg/dL per 0.1 pmol/mg increase in AF-ALB). HIV-infected people had significantly lower levels of vitamin A (-16.94 μg/dL; p <0.0001). HBV-infected people also had significantly lower levels of vitamin A (-5.66 μg/dL; p = 0.01). Multivariate regression analysis did not show a significant association between vitamin E and AF-ALB (Table 5). HIV-infected people had significantly lower vitamin E concentrations (-0.22 mg/dL).

**Table 4:** Parameter estimate of predictors associated with vitamin A

Parameter	Estimate (std err)	P value
Intercept	37.34 (3.66)	<0.0001
Aflatoxin B_1_	-4.83 (2.16)	<0.01
HIV infection	-16.94 (3.29)	<0.0001
Hepatitis B virus infection	-5.66 (2.46)	0.01
		
R^2^	0.36	
F-value	13.80	
P-value	<0.0001	

Model was adjusted for demographic variables listed in Table 1.

**Table 5:** Parameter estimate of predictors associated with vitamin E

Parameter	Estimate (std err)	P value
Intercept	0.33 (0.07)	<0.0001
Aflatoxin B_1_	-0.02 (0.04)	0.56
HIV infection	-0.22 (0.06)	<0.001
Hepatitis B virus infection	-0.007 (0.05)	0.99
		
R^2^	0.12	
F-value	3.20	
P-value	0.0007	

Model was adjusted for demographic variables listed in Table 1.

## Discussion

Our results and those of previously published studies show associations between vitamins A and E deficiencies and HIV infection [3-5]. In this study, HIV-positive individuals had higher prevalence of vitamins A and E deficiencies than HIV-negative individuals. The prevalence of vitamin A deficiency exceeded 80% in HIV-positive individuals compared with 31% among those who were HIV negative. However, the prevalence of vitamin E deficiency was generally high in both groups (88% in the HIV-positive group and 73% in the HIV-negative group), although higher in the HIV-positive group.

Although some of the foods that are high in vitamin E, such as green leafy vegetables and peanuts, are present in the diet of the study population, it is possible that there is not adequate intake of these naturally occurring sources of vitamin E. The high level of vitamin E deficiency indicates that the study participants are more likely to suffer from oxidative stress since vitamin E is an antioxidant that reduces antioxidant stress. High levels of antioxidant compounds in lymphocytes could lead to viral activation and increase in HIV viral load.

We found a significant difference in plasma AF-ALB levels between HIV-positive and HIV-negative individuals. Surprisingly, the HIV-positive individuals had higher plasma levels of AF-ALB than HIV-negative individuals. We also saw indication of impairment of liver function (AST and total protein) among HIV-positive participants. Impaired liver function has been documented in HIV-positive people [34]. Thus, HIV-positive people, probably as a result of impaired liver function, may have decreased ability to detoxify aflatoxin metabolites leading to higher concentrations of these metabolites in the blood. Aflatoxin can also cause liver disease since it induces injury to both hepatic parenchyma and the biliary tract [35]. Antiretrovirals could also induce liver toxicity in HIV-positive people on treatment [36-38].

Although we did not collect dietary information, we do not believe that the differences in AF-ALB levels between the HIV-positive and HIV-negative groups is due to whether stored or fresh grains were being eaten by a particular group. At the time that the study was conducted (June to August), both groups were likely to have been eating food stored at the end of the September to November rainy season of the previous year (harvested December to January). Participants may also have been eating some fresh food produced during the April to late June rainy season of the study year. However, because the aflatoxin albumin adduct is an indicator of aflatoxin exposure over a two- to three-month period, it is more likely that stored food is the method of exposure for both HIV-positive and HIV-negative individuals.

To the best of our knowledge, this study is the first to examine the relationship between micronutrients and aflatoxin in HIV-positive people. Almost all (99.7%) of HIV-positive study participants and all HIV-negative participants had AF-ALB in their blood. Jolly *et al *[39] have previously shown high levels of AF-ALB in a group of HIV-negative Ghanaians. We found significantly lower vitamin A concentration in study participants with high AF-ALB. Saron *et al *[40] have reported lower serum levels of retinol in individuals with chronic liver diseases, related to the severity of the condition.

Hepatic stellate cells within liver lobules store about 80% of the total body vitamin A in lipid droplets in their cytoplasm [41]. These cells also play a pivotal role in the regulation of vitamin A homeostasis [42-44]. Aflatoxin has been shown to injure both hepatic parenchyma and the biliary tract [45]. Thus, aflatoxin likely damages the liver's vitamin A functioning, and the combination of HIV and aflatoxin exacerbates the vitamin A problems faced by HIV-positive people because they have higher biological exposure.

In our study participants, HBV infection was also a strong predictor of vitamin A deficiency. Aflatoxin and HBV infection could have impacted the hepatic cells, thereby affecting vitamin A metabolism and storage. The association of vitamin A deficiency and high AF-ALB levels may result in impairment of the host immune response, which would increase susceptibility to infectious diseases and faster rate of HIV disease progression.

Vitamin E (α-tocopherol) was previously found to be positively associated with the detection rate of AFB_1_-DNA adducts in a dose-dependent manner in HBVsAg-positive and HBVsAg-negative males from Taiwan [46]. However, no association with AFB_1_-DNA adducts was found for plasma retinol. Our results revealed that aflatoxin exposure (AF-ALB) is a predictor of plasma vitamin A (retinol) status, but we did not find a significant relationship between AF-ALB and vitamin E.

The time of HIV infection was not known for our participants, and the assessment of disease progression was based on the clinical stages of the disease as determined by CD4+T cell counts and HIV viral load measurements. Changes in vitamin A status have been shown to significantly affect T cell functions in human and animal experiments [47,48]. In our study, there was no association between plasma vitamin A concentration and CD4 counts. This finding is consistent with the previous results of Jones *et al *[7], but contrary to findings by Semba *et al *[6] and Baum *et al *[49]. Although our results were not significant, we found a dose response relationship between CD4 count and vitamin A concentration. Individuals with CD4 counts <200 cells/mm^3 ^had lower vitamin A levels compared with those individuals with 200-499 cells/mm^3 ^and >499 cells/mm^3^. The lack of association between vitamin A and CD4 counts could have been confounded by the cross-sectional format of the study design. Differences in study design may explain inconsistent findings on vitamin A supplementation and HIV progression [49-51].

Vitamin E has been shown to be important in immune function [52]. Further, low serum vitamin E was found to be associated with HIV disease progression in prospective studies [49,53]. Consistent with these studies, we found a highly significant association between vitamin E and CD4 counts. Recent studies in HIV-positive people have associated vitamin E deficiency with decreased immune response, increased viral mutation and, overall, increased viral pathogenicity [11]. Beck [11] proposed that the mechanism for increased viral pathogenicity is based on the interplay between malnutrition leading to immune dysfunction, and direct oxidative damage of viral genes resulting in increased mutation rate.

Previous research has shown relationships between micronutrients and HIV viral load and between micronutrients and HIV progression [54]. We found both vitamins A and E to be significantly associated with HIV viral load; low plasma vitamin A and E levels were found in individuals with high viral load. Thus, vitamins A and E levels may be associated with HIV progression. However, the results should be interpreted with caution because our study design precludes any causal inferences about the associations. Further, the results of the study can be generalized only to people in Kumasi and its surroundings in the Ashanti Region of Ghana.

Our study permitted simultaneous assessment of several predictors of vitamins A and E, and assessment of the interaction among these predictors. In addition, we adjusted for possible confounders, such as sex and age; however, residual confounders may still have affected the study findings. There was no dietary information on the exposure to aflatoxin, but serum AF-ALB level has been shown to be a reliable biological marker of aflatoxin exposure [55]. Likewise, the study did not account for the dietary intake of vitamins A and E; therefore, it is difficult to establish that the deficiencies were caused entirely by our predictors.

Sampling all HIV-positive participants from a hospital setting and some HIV-negative participants from the community has likely introduced bias into the study. Also, we acknowledge that the p24 assay is sub-optimal for determining prevalent HIV infection. However, the HIV prevalence rate in Ghana has always been low (1.9% in 2007 and 2.2% in earlier years). Therefore, no more than about three of our potentially HIV-negative participants would have been HIV positive. Using the p24 test, we were able to rule out two potentially HIV-negative participants as HIV positive. Based on this, on participants' responses to questions regarding their health and HIV testing, and on available clinic records for HIV-negative participants who attended the clinic, we feel that it is highly likely that participants classified as HIV negative in the study were truly HIV negative.

Studies have shown relationships between aflatoxin and vitamin E; our finding of a lack of association between aflatoxin and vitamin E in the HIV-positive population might be confounded by high prevalence of vitamin E deficiency in the study group, the small sample size and the stage of HIV infection. We assumed that the variation in the time of HIV infection before enrolment is most likely random.

## Conclusions

Micronutrient deficiency and HIV infection are both major and increasingly important problems in sub-Saharan Africa. Our multivariate analysis confirms that HIV status, aflatoxin exposure and HBV infection are independent predictors of vitamin A concentration, and that HIV infection is an independent predictor of vitamin E concentration. Although we could not ascertain the effect of low vitamin A status and CD4 counts, our viral load results clearly indicate an association between vitamins A and E and HIV disease progression.

It has been found that multiple, rather than single, vitamin supplementation slows HIV progression [15]. Therefore, further studies on the association or effect of exposure to aflatoxin (and other mycotoxins) on other micronutrients in HIV-positive people are warranted so that the role of these toxins can be delineated and the appropriate steps taken to decrease exposure.

## Competing interests

The authors declare that they have no competing interests.

## Authors' contributions

FO developed the protocol, conducted vitamins A and E assays and statistical analysis, and wrote the first draft of the manuscript. PJ developed the protocol, conducted the field study, interpreted data, and revised the paper. PP assisted with protocol development and approval, participant recruitment and paper revision. AK, JE and JW reviewed the protocol, interpreted data, and participated in the revisions of the paper. YJ conducted lab analyses, interpreted lab data, and revised the paper. CP supervised vitamins A and E analysis and interpreted the data, and revised the paper. RD supervised statistical analysis, interpretation of data and revisions of the paper. All authors have read and approved the final manuscript.
